# A Rare Case of Cryptococcal Meningitis in a Child with a Congenital Heart Disease

**DOI:** 10.1155/2021/9994804

**Published:** 2021-07-29

**Authors:** Bhim Gopal Dhoubhadel, Ujjwal Laghu, Raju Poudel, Konosuke Morimoto, Koya Ariyoshi

**Affiliations:** ^1^School of Tropical Medicine and Global Health, Nagasaki University, Nagasaki, Japan; ^2^Department of Respiratory Infections, Institute of Tropical Medicine, Nagasaki University, Nagasaki, Japan; ^3^Grande International Hospital, Kathmandu, Nepal; ^4^Department of Clinical Medicine, Institute of Tropical Medicine, Nagasaki University, Nagasaki, Japan

## Abstract

Cryptococcal meningitis, which has a high mortality rate, is rare in immunocompetent children. Although many immunocompromised conditions are identified as predisposing factors, congenital heart disease in children is not well recognized as a predisposing factor for the disease. A 12-year-old female child presented with a progressively increasing headache for one month. There was history of vomiting and fever off and on. On examination, she had a pansystolic murmur and meningeal signs. Lumbar puncture was done; opening pressure was high (27 cm H_2_O) and white blood cell (WBC) count in cerebrospinal fluid (CSF) was slightly high. Gram staining, India ink preparation, and culture of CSF were negative. Cryptococcal antigen (CrAg) test was not available. Echocardiography showed a 12 mm ventricular septal defect (VSD). The patient was diagnosed as meningitis with VSD and treated with intravenous ceftriaxone and vancomycin; however, she did not improve after 7 days of antimicrobial treatment. She was then transferred to another hospital where India ink and culture of CSF showed cryptococcus; CrAg test was done, and it was positive. Despite the treatment with lyposomal amphotericin B and flucytosine, she died on 9^th^ day of admission in pediatric intensive care unit. It is necessary for global advocacy for need of availability of CrAg test in resource-limited regions. Treating doctors should consider VSD, a congenital heart disease, as a predisposing factor for cryptococcal meningitis in children. As CrAg is more sensitive than India ink and culture, it should be the first line of investigation in suspected cases.

## 1. Introduction

Cryptococcus is a common cause of meningitis among immunocompromised patients, particularly advanced immunodeficiency syndrome in adults. Although it is primarily a disease of immunocompromised adult patients, it can infect immunocompetent adults and rarely immunocompetent children. It has a high mortality rate in children [1, 2]. Cryptococcus enters into the body by inhalation from the environment, and the excreta of pigeons are the main source of infection [3].

Headache, vomiting, and mild to moderate fever are the main initial symptoms of cryptococcal meningitis (CM). Altered mental status and seizures occur in later stages of the disease, and these symptoms are more common in patients with underlying diseases [1, 4]. Fever may not be present in all cases [5, 6]. General laboratory investigations may show blood leukocytosis, elevated C-reactive protein (CRP), decreased CSF glucose, pleocytosis, increased intracranial pressure, and elevated CSF protein [6]. These findings are not specific to CM. The diagnosis is generally based on the identification of cryptococci in India ink or growth of the fungus on culture [7]. These conventional methods have limited sensitivities for the diagnosis, and culture may take many weeks; therefore, cryptococcal antigen (CrAg) test should be performed at the early stage in every meningitis patient because the survival of the patients depends on the accurate and timely diagnosis of this fatal disease [8, 9]. However, accurate and timely diagnosis and treatment remain to be a challenge in resource-limited settings [10].

Although there are various predisposing conditions for CM (Table 1), congenital heart disease (CHD) in children is not a well-known predisposing condition. Here, we report a case of CM in a child with CHD from Nepal.

## 2. Case Presentation

A 12-year-old girl presented to hospital A with a complaint of headache for one month. The headache was of sudden onset, and it gradually increased in severity over the period of time. Headache was associated with multiple episodes of vomiting. Fever was off and on. There was no history of any skin rashes, seizure, or loss of consciousness. Neither there was history of contact with a tuberculosis patient nor had she past history of any significant illnesses.

She looked ill. General examination showed heart rate 64/min, respiratory rate 30/min, temperature 37.1°C (98.7°F), and blood pressure 90/60 mm Hg. On chest examination, bilateral vesicular sounds with no added sounds were noted. Cardiovascular examination revealed normal first and second heart sounds with a pansystolic murmur. Abdomen was soft and nontender. Hepatomegaly (2 cm) was detected. No splenomegaly was detected. Glasgow comma score was 14/15. Pupils were bilateral reactive 4 mm, and the fundus showed signs of early papilledema. Examination of musculoskeletal system revealed muscle bulk was slightly decreased, tone was flaccid, and power was 4/5 in all limbs. Sensory perception was intact, tendon reflexes were grade II, and the plantar was down-going. Kernig's and Brudzinski's signs were positive. The patient was admitted with a working diagnosis of meningitis.

Investigation showed hemoglobin 13.2 g/dL (11.5–14.0 g/dL), white blood cell (WBC) count 13,000/mm^3^ (5,000–12,000/mm^3^) (neutrophil 85% (40–70%), lymphocyte 10% (28–48%), monocyte 5% (2.0–10.0%), and eosinophil 0% (2.0–6.0%)), platelets 211,000/mm^3^ (150,000–400,000/mm^3^), and erythrocyte sedimentation rate 31 mm in first hour (<20 mm). Red cell indices, liver function tests, and clotting profiles were within a normal range. Random blood sugar was 105 mg/dL (70–200 mg/dL), blood urea nitrogen was 30 mg/dL (6–20 mg/dL), and serum creatinine was 0.46 mg/dL (0.6–1.1 mg/dL). Tests for human immunodeficiency virus I and II, hepatitis B surface antigen, and hepatitis C virus were negative. Ultrasound abdomen showed mild hepatomegaly, chest X-ray showed bilateral consolidation, and MRI brain showed enhancement and mild thickening of leptomeninges without basal exudates and without hydrocephalous, features suggestive of meningoencephalitis. Echocardiograph showed perimembranous ventricular septal defect (VSD) with a size of 12 mm and was partially closed by the septal leaflet of tricuspid valve and with mild tricuspid regurgitation with normal pulmonary artery pressure.

Lumbar puncture was done. The opening pressure was 27 cm of H_2_O (7 to 20 cm of H_2_O). Results of routine and biochemistry investigation of CSF were RBC 3 cells (0 cells), WBC 204 cells (<5 cells) (neutrophil 5% (30–40%) and lymphocyte 95% (60–70%)), sugar 46 mg/dL (50–80 mg/dL), protein 24 mg/dL (15–45 mg/dL), and adenosine deaminase 2.1 U/L (<3.0 U/L). Gram stain, staining for acid-fast bacilli (AFB), India ink preparation, and culture were negative. CrAg test was not available in the hospital. Blood culture and urine culture results were negative. With these investigation findings, the patient was diagnosed with meningoencephalitis with VSD, and antimicrobial treatment was started.

The patient was treated with injection (inj) ceftriaxone 1.5 g intravenous (iv) 12 hourly and inj vancomycin 100 mg iv 6 hourly for 7 days. During hospitalization, she had one episode of seizure, which was treated by antiepileptics. She could not maintain oxygen saturation (SpO_2_) with room air, and supplemental oxygen was provided. After 7 days of the antimicrobial treatments, her general condition did not improve. She was then brought to hospital B.

**Figure 1:**
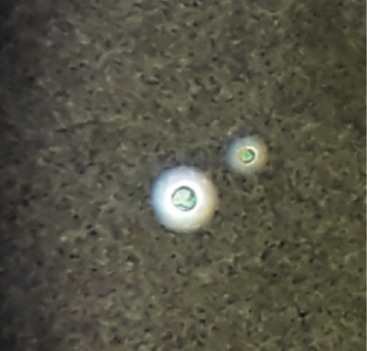
India ink preparation in CSF sample showing the capsule (a zone of clearance or “halo”) that surrounds the yeast cells.

**Figure 2:**
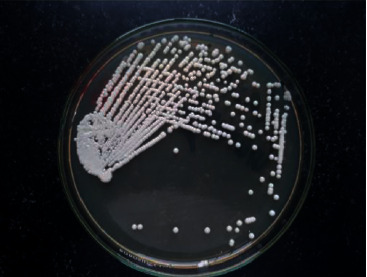
Culture of CSF showing white smooth colonies of *Cryptococcus neoformans* in Sabouraud dextrose agar.

At the time of admission to hospital B, she was at semiconscious state. She was admitted to the pediatric intensive care unit. Further laboratory investigations: leptospira immunoglobulin M, brucella immunoglobulin M, malaria antigens, and Mantoux tuberculin skin tests were done, and all came out negative. Blood culture and urine culture were negative. Sputum AFB and culture were negative. GeneXpert was not available. It was important to rule out tubercular meningitis. There was no history of contact with active tubercular patients, CSF color was clear, CSF AFB staining was negative, and CSF ADA was not raised. Therefore, we did not consider tubercular meningitis as the first differential diagnosis. CSF India ink preparation showed *Cryptococcus* spp. (5 to 10 *μ*M in size) with surrounding with a capsule (Figure 1). CSF culture, Sabouraud dextrose agar media, after 48 hours showed whitish mucoid colonies (Figure 2). CrAg test in CSF by enzyme immunoassay was positive. CM was diagnosed and treated with inj lysosomal amphotericin B 100 mg in 750 ml 5% dextrose iv over 2 hours once a day and inj flucytosine 625 mg in 50 ml iv 6 hourly.

The general condition of the patient did not improve despite the treatment for CM. She had one episode of seizure, which was treated with antiepileptics. She developed severe respiratory distress; she was intubated and kept on mechanical ventilation. On 9th day of admission, she died.

## 3. Discussion

Here, we presented a fatal case of CM in a 12-year-old girl with congenital heart disease. Congenital heart disease is not a well-recognized predisposing condition for cryptococcal disease. This case highlights a picture of delayed diagnosis and poor outcome of CM in a resource-limited region. The girl had headache for a month when presenting to the first hospital. She was investigated for suspected meningoencephalitis by examination for CSF. Due to low sensitivity of India ink preparation and culture, identification of cryptococcus was missed and treatment delayed for many days. Repeated investigations for cryptococcus were not performed in the first hospital, and a more sensitive CrAg test was also not available there. As the chest radiograph showed a bilateral consolidation, we believe that the patient had cryptococcal pneumonia at the beginning although the sputum culture and AFB were negative; the infection later spread to the brain, which might be predisposed by the VSD.

Immunocompromised conditions are the common predisposing factors for CM; however, it can infect the immunocompetent host, irrespective of age. It is prevalent among men of age 30 to 50 years [1, 11]. Although CM is rare in children, it has high mortality rate in this age group [1, 2, 11]. It affects more male children and children from rural areas [6]. Children who had contact with birds or bird droppings were more likely to be affected (OR: 11.8; 95% CI: 2.2–62.2) and children who presented with onset of symptoms of more than 20 days at risk of the disease (OR: 5.3; 95% CI: 1.6–17.9) [6]. Although many predisposing conditions for CM are known (Table 1), congenital heart disease is not well recognized as a predisposing factor in children. Similarly, many children who have CM do not have any identifiable diseases; this implies that we should not forget to test for this disease even though children do not have any underlying diseases [4].

CM and other causes of meningitis have similar clinical features as well as laboratory findings in CSF routine and biochemistry tests; therefore, it is difficult to differentiate CM from tubercular, bacterial, or viral meningitis [12]. In resource-limited regions, the diagnosis of CM depends entirely on identification of cryptococcus on CSF India ink smear or fungal culture, and detection of specific antigen by cryptococcal antigen tests may not be available. As the sensitivity of India ink smear or fungal culture is limited, many CM are missed and misdiagnosed as other diseases. Tubercular meningitis is the most frequent misdiagnosis [4, 13]. The survival of these CM patients depends on an accurate diagnosis, which can be enhanced by the provision of availability of CrAg test. CrAg is a life-saving diagnostic test in resource-limited regions, and its availability in these regions needs an urgent global advocacy [10].

Cryptococcus is an encapsulated yeast. On India ink microscopy, it is detected by the nonstaining capsule around the organism. It can be mistaken for lymphocytes or other mononuclear cells if nonstaining capsule is not distinguished [7]. This is a simple and easily available test in resource-limited regions; unfortunately, its sensitivity is 42% when the CSF cryptococcus density is lower than 1000 CFU/mL [14]. CSF centrifugation can possibly increase the sensitivity, but it is still lower than CrAg detection test [14]. CSF culture and growth of cryptococcus on Sabouraud medium can take days or weeks for the definitive results [7, 14]. Although it is considered as the gold standard for the definite diagnosis, lack of availability of culture facility in resource-limited regions, longer time for incubation for diagnosis, and lower sensitivity are major disadvantages [14].

CrAg can be identified by latex agglutination test, and its sensitivities and specificities are 93–100% and 93–98%, respectively, for CSF specimens [14, 15]. In our patient, this test was done only in the second hospital. If this test were available in the first hospital, it would be possible that the child could be diagnosed earlier, and the necessary treatment could be started earlier. CrAg lateral flow assay is an immunochromatic dipstick test that also detects CrAg, and it has high sensitivity (99.3%) and specificity (99.1%) for diagnosing CM [9]. This dipstick test is suitable for resource-limited regions as well as for the field surveillance as it does not require laboratory infrastructure that includes the refrigeration. Serum, plasma, or CSF sample can be used, and the results are available after 10 min [9]. Therefore, this test along with India ink are highly recommended for screening of CM in every meningoencephalitis patient to ensure the earliest diagnosis [8].

Diagnosis and treatment of cryptococcal infection remains to be a challenge in resource-limited regions, and it affects the survival of the patients [10]. CM is most prevalent in sub-Saharan Africa where HIV is prevalent. World Health Organization recommends a lumbar puncture with measurement of CSF opening pressure and CrAg test as preferred diagnostic method in these regions. When a lumbar puncture cannot be performed, the antigen assays should be tested on serum, plasma, or whole blood [16]. Despite the WHO recommendation, there is still lack of availability of CrAg tests in hospitals and health centers in resource-limited regions [10].

In conclusion, CM needs to be considered as a differential diagnosis in all cases of meningoencephalitis, including seemingly immunocompetent children. Congenital heart disease can be a predisposing factor. CrAg test should be performed as early as possible so that necessary treatment can be started on time to prevent the impending death; however, this highly sensitive test is not available in many resource-limited regions.

## Figures and Tables

**Table 1:** Predisposing conditions for cryptococcal meningitis [4, 11, 12].

Known predisposing conditions
HIV/AIDS
Hematological cancer
Prolonged corticosteroids therapy
Diabetes
Nephrotic syndrome
Henoch-Schonlein purpura
Idiopathic thrombolytic purpura
Juvenile rheumatoid arthritis
Tuberculosis
Alcoholism
Sarcoidosis
Systematic lupus erythematous
Still's disease
Chronic hepatitis
X-linked agammaglobulinemia

## Data Availability

Data related to this study can be obtained upon a reasonable request to the corresponding author.

## Abbreviations

CHD: Congenital heart disease
CM: Cryptococcal meningitis
CrAg: Cryptococcal antigen
CSF: Cerebrospinal fluid
LP: Lumbar puncture
VSD: Ventricular septal defect
WBC: White blood cell.
